# Increasing LH Pulsatility in Women With Hypothalamic Amenorrhoea Using Intravenous Infusion of Kisspeptin-54

**DOI:** 10.1210/jc.2013-1569

**Published:** 2014-02-11

**Authors:** Channa N. Jayasena, Ali Abbara, Johannes D. Veldhuis, Alexander N. Comninos, Risheka Ratnasabapathy, Akila De Silva, Gurjinder M. K. Nijher, Zainab Ganiyu-Dada, Amrish Mehta, Catriona Todd, Mohammad A. Ghatei, Stephen R. Bloom, Waljit S. Dhillo

**Affiliations:** Section of Investigative Medicine (C.N.J., A.A., A.N.C., R.R., A.D.S., G.M.K.N., Z.G.-D., M.A.G., S.R.B., W.S.D.), Imperial College London, Hammersmith Hospital, London W12 ONN, United Kingdom; and Endocrine Research Unit (J.D.V.), Center for Translational Science Activities, Mayo Clinic, Rochester, Minnesota 55905; and Department of Imaging (A.M., C.T.), Imperial College Healthcare National Health Service Trust, Charing Cross Hospital, London W6 8RF, United Kingdom

## Abstract

**Background:**

Hypothalamic amenorrhea (HA) is the one of the most common causes of period loss in women of reproductive age and is associated with deficient LH pulsatility. High-dose kisspeptin-54 acutely stimulates LH secretion in women with HA, but chronic administration causes desensitization. GnRH has paradoxical effects on reproductive activity; we therefore hypothesized that a dose-dependent therapeutic window exists within which kisspeptin treatment restores the GnRH/LH pulsatility in women with HA.

**Aim:**

The aim of the study was to determine whether constant iv infusion of kisspeptin-54 temporarily increases pulsatile LH secretion in women with HA.

**Methods:**

Five patients with HA each underwent six assessments of LH pulsatility. Single-blinded continuous iv infusion of vehicle or kisspeptin-54 (0.01, 0.03, 0.10, 0.30, or 1.00 nmol/kg/h) was administered. The LH pulses were detected using blinded deconvolution.

**Results:**

Kisspeptin increased LH pulsatility in all patients with HA, with peak responses observed at different doses in each patient. The mean peak number of pulses during infusion of kisspeptin-54 was 3-fold higher when compared with vehicle (number of LH pulses per 8 h: 1.6 ± 0.4, vehicle; 5.0 ± 0.5, kisspeptin-54, *P* < .01 vs vehicle). The mean peak LH pulse secretory mass during kisspeptin-54 was 6-fold higher when compared with vehicle (LH pulse secretory mass in international units per liter: 3.92 ± 2.31, vehicle; 23.44 ± 12.59, kisspeptin-54; *P* < .05 vs vehicle).

**Conclusions:**

Kisspeptin-54 infusion temporarily increases LH pulsatility in women with HA. Furthermore, we have determined the dose range within which kisspeptin-54 treatment increases basal and pulsatile LH secretion in women with HA. This work provides a basis for studying the potential of kisspeptin-based therapies to treat women with HA.

Hypothalamic amenorrhea (HA) is a heterogeneous condition characterized by an acquired reduction in GnRH secretion, which is reflected by reduced basal circulating LH levels (1, 2) and reduced frequency/amplitude of LH pulsatility (3, 4). HA is one of the most common causes of period loss in women of reproductive age (5) and has been observed in 10% of female athletes in a previous study (6). Affected patients may have one or more etiological factors including low body weight, excessive exercise, and stress (7). Furthermore, heterozygous mutations in genes associated with congenital GnRH deficiency have been identified in patients with HA (8). Estrogen supplementation provides women with HA with symptomatic relief and protection from osteoporosis. However, restoration of fertility in HA requires restoration of hypothalamo-pituitary function, which is not provided by estrogen supplementation. HA patients with poor functional estrogen status assessed by progestin testing (9) respond poorly to clomiphene citrate, which reduces estradiol-mediated negative feedback (10). In vitro fertilization is highly labor intensive, and treatment with recombinant FSH alone may stimulate inadequate estradiol production in patients with GnRH deficiency (11). GnRH sc pulsatile administration is also an alternative therapeutic for HA with much lower risks of multiple pregnancy when compared with in vitro fertilization (12, 13). It is therefore important to develop novel therapies to restore hypothalamo-pituitary function in patients with HA.

A hallmark of HA is loss of the pulsatile pattern of GnRH/LH secretion, which is normally observed in healthy subjects. Seminal experiments by Knobil et al (14) paved the way to our understanding that GnRH administration must be intermittent and pulsatile to restore activity of the reproductive axis in patients with HA (and other disorders of GnRH deficiency); furthermore, tonic exposure to GnRH paradoxically inhibits pituitary gonadotrophin secretion in healthy subjects (15). The physiological pulsatile pattern of GnRH secretion (and therefore LH secretion) is tightly regulated by a neurohumoral network termed the GnRH pulse generator (16). The hypothalamic neuropeptide kisspeptin is a recently identified component of the GnRH pulse generator. Loss of function mutations in genes encoding kisspeptin or its receptor causes pubertal failure, hypogonadotropic hypogonadism, and loss of fertility in humans (17–19). Administration of kisspeptin potently stimulates gonadotropin secretion in all mammalian species tested to date, an effect that is abolished by pretreatment with a GnRH antagonist (20–26). Kisspeptin increases the rate of electrical firing in GnRH neurons in vitro (27). Conversely, pharmacological kisspeptin antagonism reduces GnRH and LH pulsatility in monkeys and sheep, respectively (28). These data therefore suggest that endogenous hypothalamic kisspeptin may positively regulate GnRH/LH pulsatility. Kisspeptin is therefore a potential therapeutic for restoring activity of the reproductive axis in patients with HA.

We have recently demonstrated that peripheral administration of the full-length kisspeptin peptide, kisspeptin-54, potently stimulated gonadotrophin secretion in healthy men and women (29, 30). We have also observed that administration of high doses of kisspeptin-54 acutely and potently stimulates gonadotropin secretion in women with HA but chronically causes tachyphylaxis (desensitization to its effects) (31). Considering the historical studies of GnRH, we hypothesized there may exist a dose-dependent therapeutic window within which kisspeptin treatment restores the GnRH/LH pulsatility needed for fertility in women with HA.

We conducted a placebo-controlled, single-blinded study to determine whether constant iv infusion of kisspeptin-54 temporarily restored pulsatile LH secretion in women with HA.

## Materials and Methods

### Subjects

Ethical approval was granted by the local ethics research committee (registration number 05/Q0406/142). Written informed consent was obtained from all subjects. This study was performed in accordance with the Declaration of Helsinki. Five newly diagnosed patients with HA were recruited through advertisements placed in local newspapers. Responders to adverts were evaluated with a detailed menstrual history, clinical examination, and blood tests as follows: full blood count, renal profile, liver profile, bone profile, glucose, thyroid profile, LH, FSH, estradiol, progesterone, androstenedione, dehydroepiandrosterone, T, SHBG, prolactin, 17-hydroxyprogesterone, and cortisol. Women were diagnosed with functional HA and included within the study if they fulfilled the following criteria: body mass index less than 25 kg/m^2^, stable body weight over the previous 6 months, age between 18 and 40 years, secondary amenorrhea of at least 6 months' duration, absence of oral contraceptive pill therapy for at least 1 year, absence of systemic disease comorbidity, absence of active psychiatric illness including anorexia nervosa or other eating disorders, absence of therapeutic or recreational drug use, absence of clinical or biochemical hyperandrogenemia, structurally normal hypothalamo-pituitary region assessed by magnetic resonance imaging, no clinical or biochemical evidence of polycystic ovarian syndrome, normal thyroid function tests, normal serum prolactin levels, and serum LH to FSH ratio less than 1.5. Baseline patient characteristics are summarized in Table 1.

**Table 1.** Baseline Characteristics of Participants With HA

Participant	1	2	3	4	5
Age, y	30.1	30.3	24.2	28.3	31.3
Weight, kg	47.8	40.6	51.8	59.8	49.8
Body mass index, kg/m^2^	15.4	17.3	17.6	20.6	19.0
Duration of amenorrhea, mo	60	7	9	32	7.0
Serum LH, IU/L	<0.5	0.6	0.7	0.6	2.5
Serum FSH, IU/L	1.2	6.5	4.8	3.8	5.0
Serum E2, pmol/L	<70	192	<70	<70	<70

### Kisspeptin-54 peptide

Kisspeptin-54 was synthesized, purified, and tested as previously described (23–25). Vials of freeze-dried saline or kisspeptin were stored at −20°C reconstituted in 0.5 mL of 0.9% saline. The doses selected during this study were based on our previous data on the effects of kisspeptin in women with HA (31, 32).

### Study protocol

Each patient with HA underwent six single-blinded assessments of LH pulsatility, which were performed in a random order. Study visits for individual patients were scheduled a minimum of a week apart. Patients were admitted to our clinical investigation unit at 8:00 am and asked to lay supine. Urine was tested to exclude pregnancy (Clearview easy-HCG; Inverness Medical Innovations Inc). A single-blinded continuous iv infusion of gelofusin vehicle or kisspeptin-54 (0.01, 0.03, 0.10, 0.30, or 1.00 nmol/kg/h) was commenced at time 0 minutes, lasting for 8 hours except during the administration of the highest dose of kisspeptin-54 (1 nmol/kg/h), when the administration lasted for 10 hours. A 2-fold higher infusion rate was administered during the first 30 minutes of each infusion to achieve steady-state plasma levels. Blood samples were taken through a cannula at 10-minute intervals for the entire duration of infusion of gelofusin or kisspeptin. Two GnRH tests were performed in patients with HA: immediately after stopping infusion of the highest dose of kisspeptin-54 (1 nmol/kg/h) and 1 week after stopping 1 nmol/kg/h kisspeptin-54. Each GnRH test consisted of a 100-μg iv bolus of GnRH followed by blood sampling for 2 hours. Blood samples for serum and plasma analysis were collected as previously described (29–31, 33).

### Analytical methods

Serum LH, FSH, and estradiol levels were measured using automated chemiluminescent immunoassay (Abbott Diagnostics) using the Second International Pituitary LH Standard (80/552). Reference ranges were as follows: LH in international units per liter, 2–10 (follicular), 20–60 (midcycle), 4–14 (luteal); FSH in international units per liter, 1.5–8 (follicular and luteal), 10–50 (midcycle); and estradiol (E2) in picomoles per liter, less than 300 (early follicular), 400–1500 (midcycle), 200–1000 (luteal). The respective intraassay and interassay coefficients of variation for each assay were as follows: 4.1% and 2.7% (LH); 4.1% and 3.0% (FSH); and 3.3% and 3.0% (E2). Analytical sensitivities were as follows: 0.5 IU/L (LH), 0.05 IU/L (FSH); and 37 pmol/L (E2). Measurement of plasma kisspeptin immunoreactivity (IR) was performed using an established RIA (29–31, 33).

### Data analysis

Data are presented as mean ± SEM. Multiple means were compared using one-way ANOVA with Tukey's post hoc analysis. *P* < .05 was considered statistically significant. J.D.V. used a previously described, blinded deconvolution method with 93% sensitivity and specificity to analyze LH pulsatility (34). Specifically, the number of LH pulses, secretory mass of each LH pulse, and estimate levels of basal and pulsatile LH secretion were analyzed during each study visit.

## Results

### Kisspeptin IR in plasma was raised after injection of kisspeptin

**Figure 1.**
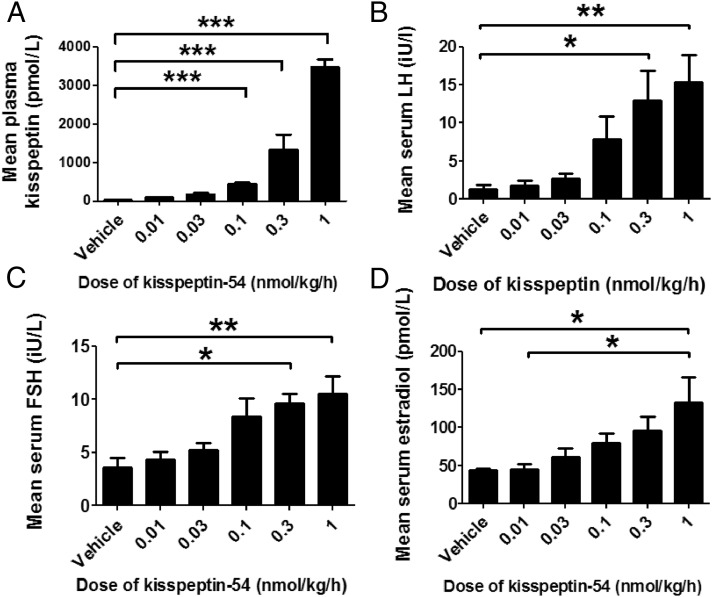
Mean circulating levels of kisspeptin and other reproductive hormones during an infusion of kisspeptin-54 to women with HA. Five women with HA each underwent an iv infusion of vehicle or kisspeptin-54 (0.01, 0.03, 0.1, 0.3, or 1.0 nmol/kg/h). The duration of the infusion was 480 minutes except at the 1.0 nmol/kg/h kisspeptin-54 dose, which lasted 600 minutes. Data presented are mean ± SEM for Plasma kisspeptin IR in picomoles per liter (A), serum LH in international units per liter (B), serum FSH in international units per liter (C), and serum E2 in picomoles per liter (D). *, *P* < .05; **, *P* < .01; ***, *P* < .001.

Mean plasma kisspeptin IR was barely detectable (<10 pmol/L) during the 8-hour period during infusion of vehicle (Figure 1A). Plasma kisspeptin IR was markedly elevated during the infusion of the three highest doses of kisspeptin-54 (0.10, 0.30, and 1.00 nmol/kg/h) when compared with infusion of vehicle (*P* < .001 vs vehicle at each dose). Mean plasma kisspeptin IR during infusion of 1.00 nmol/kg/h kisspeptin-54 was 3469 ± 208 pmol/L, which is comparable with levels of kisspeptin-IR observed during human pregnancy (35).

### Effects of kisspeptin infusion on mean levels of serum LH, FSH, and E2

As expected, the mean levels of LH, FSH, and E2 secretion were very low in patients with HA during the administration of vehicle. A trend of increasing mean levels of LH, FSH, and E2 was observed during increasing doses of kisspeptin-54 infusion in patients with HA (Figure 1, B–D). Mean serum LH was increased greater than 10-fold during an infusion of the highest dose of kisspeptin-54 when compared with vehicle in patients with HA (mean level of serum LH during infusion: 1.26 ± 0.56, vehicle; 15.42 ± 3.57, 1.00 nmol/kg/h kisspeptin-54, *P* < .01 vs vehicle). Mean levels of serum FSH and E2 were also increased significantly during an infusion of the highest dose of kisspeptin-54 when compared with vehicle in patients with HA (Figure 1, C and D).

### Effect of kisspeptin infusion on LH pulsatility

#### Number of LH pulses

**Figure 2.**
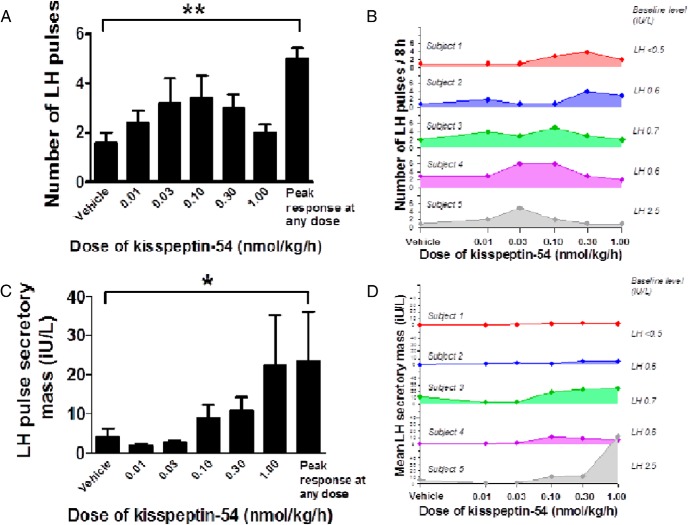
Effects of kisspeptin-54 on LH pulsatility in women with HA. A and C, Mean number of LH pulses (A) and LH pulse secretory mass (C) are presented for study visits during an iv infusion of vehicle or kisspeptin-54 (0.01, 0.03, 0.1, 0.3, or 1.0 nmol/kg/h). Peak responses to kisspeptin-54 were observed at different doses in each patient. We therefore also examined the peak number of LH pulses and peak secretory mass during any dose of kisspeptin-54 in each patient and present a mean value in all five subjects: peak response at any dose. Mean ± SEM of the number of pulses and pulse secretory mass are presented over 480 minutes except for the 1.0 nmol/kg/h dose, which was extended to 600 minutes. *, *P* < .05; **, *P* < .01. B and D, Dose response of the number of LH pulses (B) and LH pulse secretory mass (D) in each individual subject during an iv infusion of vehicle or each dose kisspeptin-54.

As expected, very few LH pulses (1.6 ± 0.4 per 8 h) were observed during the infusion of vehicle in women with HA (Supplemental Figure 1, published on The Endocrine Society's Journals Online web site at http://jcem.endojournals.org, and Figure 2A). Nonsignificant increases in number of LH pulses were observed during the infusion of kisspeptin-54 when compared with vehicle, particularly at the doses 0.03 and 0.10 nmol/kg/h. Examining the response of individual patients to kisspeptin-54, it appeared that peak numbers of LH pulses were observed at different doses in each patient with HA (Figure 2B); the mean peak number of pulses (at any dose of kisspeptin-54) was 3-fold higher when compared with vehicle in patients with HA (number of LH pulses per 8 h: 1.6 ± 0.4, vehicle; 5.0 ± 0.5, dose of kisspeptin-54 associated with peak number of pulses, *P* < .01 vs vehicle) (Figure 2A).

#### LH secretory mass

We then examined the mean levels of LH secretory mass during the infusion of kisspeptin-54 to patients with HA (Figure 2C). Mean LH secretory mass increased nonsignificantly during the three highest doses of kisspeptin-54 (0.10, 0.30, and 1.00 nmol/kg/h) when compared with vehicle. There appeared to be considerable variation between patients in the levels of LH pulse secretory mass during high doses of kisspeptin-54 infusion (Figure 2D). Furthermore, peak levels of LH pulse secretory mass were observed at different doses in each patient with HA (Figure 2D). The mean peak LH secretory mass (at any dose of kisspeptin-54) was 6-fold higher when compared with vehicle in patients with HA (LH pulse secretory mass in international units per liter: 3.92 ± 2.31, vehicle; 23.44 ± 12.59, dose of kisspeptin-54 associated with peak secretory mass; *P* < .05 vs vehicle) (Figure 2C).

#### Estimated total levels of basal and pulsatile LH secretion

We also calculated total levels of basal and pulsatile LH secretion during each study visit in patients with HA (Supplemental Figure 2). Consistent with the mean serum LH levels, basal LH secretion increased in a dose-dependent manner during the infusion of kisspeptin-54 in patients with HA (Supplemental Figure 2, A and B). The total levels of pulsatile LH secretion are dependent on both frequency and secretory mass of LH pulses. Pulsatile LH secretion was unchanged during the two lowest doses of kisspeptin-54 infusion when compared with vehicle (Supplemental Figure 2, C and D). However a 4-fold increase in the mean pulsatile LH secretion was observed during 0.10 nmol/kg/h kisspeptin-54 when compared with the vehicle (mean pulsatile LH in international units per liter: 7.0 ± 4.3, vehicle; 37.9 ± 17.7, dose of kisspeptin-54 associated with peak number of pulses; *P* < .05 vs vehicle). Increases in mean pulsatile LH secretion were also observed during 0.30 and 1.00 nmol/kg/h kisspeptin-54 when compared with vehicle in patients with HA, but these increases were nonsignificant.

### Desensitization during high-dose infusion of kisspeptin-54

#### Plasma kisspeptin

**Figure 3.**
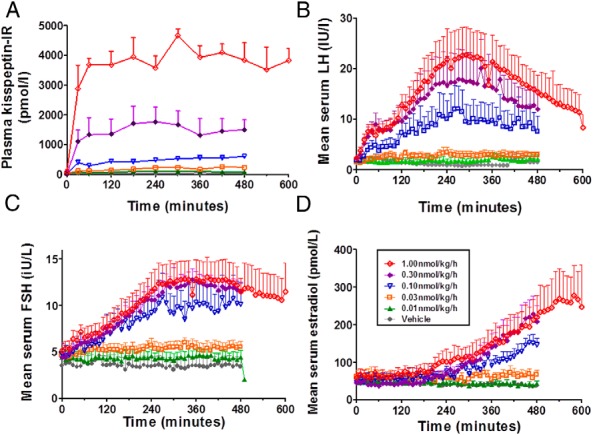
Time profiles of circulating kisspeptin and other reproductive hormones during an infusion of kisspeptin-54 to women with HA. Five women with HA each underwent an iv infusion of saline vehicle (gray) or 0.01 (green), 0.03 (orange), 0.10 (blue), 0.30 (purple), or 1.00 (red) nmol/kg/h kisspeptin-54. between 0 and 480 minutes (except a 600 min duration with 1.0 nmol/kg/h kisspeptin-54) commencing at 0 minutes. Data presented are mean ± SEM for plasma kisspeptin IR in picomoles per liter (A), serum LH in international units per liter (B), serum FSH in international units per liter (C), and serum E2 in picomoles per liter (D). *, *P* < .05; **, *P* < .01; ***, *P* < .001.

Levels of plasma kisspeptin reached plateau concentrations 1 hour after commencing kisspeptin-54 infusion in women with HA and remained steady until the end of the infusion (Figure 3A).

#### Serum LH

**Figure 4.**
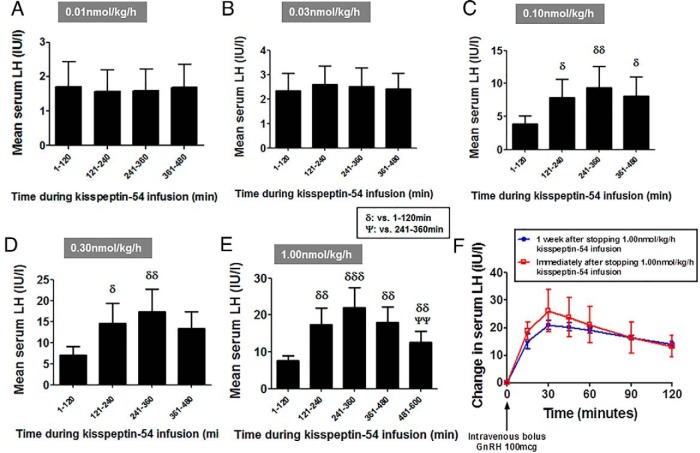
High-dose infusion of kisspeptin-54 is associated with tachyphylaxis in women with HA. A–E, Mean levels of serum LH during each 120-minute time period during an iv infusion of kisspeptin-54. For each 120-minute time period, mean values presented in the figure were derived from a single mean level of serum LH in each subject for each 120-minute time period. Duration of sampling was for 480 minutes except for the 1.0 nmol/kg·h dose, which was extended for 600 minutes. Data are mean ± SEM. δ, *P* < .05 vs 1–120 minutes; δδ, *P* < .01 vs 1–120 minutes; δδδ, *P* < .001 vs 1–120 minutes; ψψ, *P* < .01 vs 241–360 minutes. F, To investigate the desensitization in LH response observed during an infusion of 1.00 nmol/kg·h kisspeptin-54, the pituitary response to an iv bolus GnRH (100 μg) was assessed immediately after and 1 week after the cessation of 1.00 nmol/kg·h kisspeptin-54. Data are presented as mean ± SEM for change in LH in international units per liter after the administration of GnRH.

No significant desensitization to kisspeptin-54 was observed in LH secretion during 0.01 to 0.30 nmol/kg/h kisspeptin-54 infusion (Figures 3B and 4, A–D). At the highest dose of kisspeptin-54 infusion (1.00 nmol/kg/h), the serum LH levels peaked approximately 5 hours after commencing infusion and then gradually decreased thereafter (Figure 3B); the mean levels of serum LH during the final 2 hours (481–600 min) of 1.00 nmol/kg/h kisspeptin-54 were significantly lower when compared with the fourth to fifth hours of infusion (241–300 min) (mean serum LH in international units per liter: 22.03 ± 5.42, 241–360 min; 12.57 ± 3.05, 481–600 min; *P* < .01 vs 241–360 min) (Figure 4E). To assess whether desensitization to kisspeptin was occurring at the level of the pituitary gland, GnRH tests were performed immediately after the infusion of 1.00 nmol/kg/h kisspeptin-54 and 1 week after stopping the infusion of 1.00 nmol/kg/h kisspeptin-54 (Figure 4F); the mean LH secretion after the GnRH bolus was almost identical on both occasions, which suggested that desensitization to kisspeptin-54 did not involve the pituitary gland.

#### Serum FSH and estradiol

No significant desensitization to kisspeptin-54 was observed in FSH or E2 secretion during the lower doses of kisspeptin-54 infusion (Figure 3, C and D). Indeed, levels of serum E2 continued to increase gradually during the infusion of 1.00 nmol/kg/h kisspeptin-54, despite contemporaneous desensitization in LH response to kisspeptin-54 at this dose (Figure 3D).

## Discussion

Our data suggest for the first time that constant iv infusion of kisspeptin-54 may temporarily increase both basal and pulsatile LH secretion in women with HA. Furthermore, we have observed that LH pulsatility may be stimulated in HA at doses, which are distinct to the doses associated with desensitization during iv kisspeptin-54 infusion.

The hypothalamic neuropeptide kisspeptin is essential for pubertal activation in humans (21, 22). Kisspeptin potently stimulates gonadotropin secretion, an effect that is abolished by the pretreatment with a GnRH antagonist (20–26). We may therefore infer that peripheral kisspeptin stimulates LH through a GnRH-dependent mechanism. Kisspeptin receptors have been identified on GnRH nerve terminals in rodent median eminence, an area of the hypothalamus with an incomplete blood-brain barrier (36). Furthermore, recent tracer studies highlight that kisspeptin neurones in the goat arcuate nucleus project to these GnRH nerve terminals (37). If these findings are also true in humans, the peripheral administration of kisspeptin-54 may act on the GnRH nerve terminals to stimulate endogenous GnRH secretion.

It is important to recognize that although our data suggest that kisspeptin-54 increases LH pulsatility in women with HA, we cannot assume that kisspeptin-54 is stimulating endogenous GnRH pulsatility. It is possible that peripheral kisspeptin-54 acts at the GnRH nerve terminals within the median eminence to amplify preexisting, undetectable GnRH/LH pulses into detectable GnRH/LH pulses in women with HA. An alternative explanation of our data is that exogenous, peripheral kisspeptin-54 stimulates de novo GnRH pulsatile secretion through a direct or indirect mechanism. Recent studies by Chan et al (38) demonstrated that a single injection of the short form of kisspeptin, kisspeptin-10, elicited a pharmacological LH pulse, which was distinct from the preexisting, endogenous LH pulses in healthy men. However, prolonged infusion of kisspeptin-10 increases the number of LH pulses in healthy men and patients with inactivating mutations in the neurokinin B signaling pathway (39, 40). Our immunoreactivity data suggest that kisspeptin exposure was constant during kisspeptin-54 infusion. It is therefore possible that prolonged exposure to exogenous kisspeptin is required to increase LH pulsatility in patients with HA.

Pulsatile administration of GnRH is another treatment for HA, which can restore fertility in affected patients (12, 41). Pulsatile GnRH treatment is clinically available in only a few countries, possibly due to the limited commercial availability of portable pump devices capable of pulsatile administration. It is premature to consider kisspeptin-54 as a therapeutic for HA; however, one potential advantage of administering kisspeptin-54 over GnRH would be its suitability for constant pump administration, which is increasingly used to deliver insulin therapy to certain patients with diabetes (42). Further studies would be needed to compare the effectiveness of kisspeptin-54 and pulsatile GnRH administration for patients with HA.

We have previously observed that high sc doses of kisspeptin 54 acutely and potently stimulated gonadotropin secretion in women with HA but chronically led to tachyphylaxis (desensitization to its effects) (31, 32). We therefore hypothesized there may exist a dose-dependent therapeutic window within which kisspeptin treatment restored LH pulsatility in women with HA, without causing tachyphylaxis. Mean serum LH increased dramatically after initiating 1.00 nmol/kg/h kisspeptin-54 infusion, to peak levels 5h after infusion commencement; thereafter, levels of mean serum LH decreased gradually to levels which were 50% lower by the end of the 10h infusion when compared with peak levels. It is interesting to consider whether sex-steroid feedback contributed to this fall in gonadotrophin response to 1.00 nmol/kg/h kisspeptin-54 infusion. Seminara et al (43) observed that desensitization occurred within 3 hours of commencing kisspeptin-10 infusion within an agonadal monkey model. Furthermore, we previously observed that desensitization to chronic kisspeptin-54 treatment occurred in HA patients without any significant elevation in circulating E2 levels (31). On balance, we consider that tachyphylaxis is the most likely explanation for falling gonadotrophin levels during 1.00 nmol/kg/h kisspeptin-54 infusion; however, we cannot exclude that sex steroid feedback was a contributory factor in our gonadal-intact model.

Energy availability is an important determinant of reproductive function; disturbances in LH pulsatility during a controlled exercise regimen are predominantly observed if female subjects simultaneously undergo restriction of caloric intake (44). The adipokine hormone leptin is a key mediator of energy availability. Low levels of circulating leptin are observed in women with HA when compared with weight-matched controls (45, 46). Furthermore, previous studies have demonstrated that chronic treatment with recombinant leptin restores LH pulsatility in some, but not all, patients with HA (47). In view of the small sample size of our study, it is important to acknowledge that a number of etiological factors may cause HA. It is therefore likely that a distinct combination of precipitants such as leptin deficiency, weight loss, excessive exercise, stress, and mutations of GnRH-associated genes caused HA in each of our five participants included in our study; these etiological differences may have partially accounted for heterogeneity of individual responses to kisspeptin-54 administration. Interpretation of study results should also be qualified by considering that the body mass index criterion less than 25 kg/m^2^ may have skewed subject selection toward nutritional causes of HA. Furthermore, we did not assess the functional estrogen status of our participants using progestin testing or endometrial thickness measurement during ultrasonography. We recognize that a range of functional estrogen status may have been present in our study cohort and may have contributed to the heterogeneity of individual responses to kisspeptin-54 administration.

The LH pulsatility data during high dose kisspeptin-54 infusion are of particular interest. Significant LH pulses were detected during, but not after, the initial 4 hours of high-dose kisspeptin-54 administration. As discussed previously, serum levels of LH began to fall around this time point due to desensitization to kisspeptin-54. It is therefore possible that tachyphylaxis to kisspeptin-54 may have inhibited pulsatile LH secretion in women with HA. It is also noteworthy that LH pulses detected during the high-dose kisspeptin infusion were calculated to have a much higher mean secretory mass when compared with a saline infusion. Two explanations for this observation may be considered when inspecting the individual LH profiles (Supplemental Figure 1). First, it is possible that high-dose kisspeptin-54 stimulates endogenous pulsatile LH secretion, leading to high-amplitude LH pulses in addition to rising levels of basal LH secretion. There is a biological precedent for this observation: frequent blood sampling every 2–3 minutes reveals that the rat proestrous LH surge is associated with a 20-fold increase in pulsatile LH secretion in addition to rising levels of basal LH secretion (48). A second explanation for observing high-amplitude pulses during high-dose kisspeptin-54 is that detected LH pulses were pharmacological rather than endogenous in origin. In support of this, iv bolus kisspeptin-10 stimulates a single pharmacological LH pulse in healthy men and women (38). More detailed blood sampling every 2–5 minutes may help to resolve whether detected LH pulses during high dose kisspeptin-54 administration to women with HA are endogenous (ie, distinct to basal LH secretion) or pharmacological in origin.

In summary, we demonstrate for the first time that infusion of kisspeptin-54 temporarily stimulates LH pulsatility in a common form of female infertility. In particular, we have determined the dose range within which kisspeptin-54 treatment restores basal and pulsatile LH secretion in women with HA. This work provides a basis for studying the potential of kisspeptin-based therapies to treat women with HA.
